# Draft genomes of *Lactobacillus delbrueckii* and *Klebsiella pneumoniae* coexisting within a female urinary bladder

**DOI:** 10.1128/MRA.00305-23

**Published:** 2023-09-22

**Authors:** Laricca Y. London, Chae Hee Lim, Jennifer L. Modliszewski, Nazema Y. Siddiqui, Tatyana A. Sysoeva

**Affiliations:** 1 Department of Biological and Environmental Sciences, Alabama A&M University, Normal, Alabama, USA; 2 Department of Biological Sciences, The University of Alabama in Huntsville, Huntsville, Alabama, USA; 3 Department of Bioinformatics and Biostatistics, Duke University Center for Genomic and Computational Biology, Duke University, Durham, North Carolina, USA; 4 Department of Obstetrics and Gynecology, Division of Urogynecology and Reconstructive Pelvic Surgery, Duke University, Durham, North Carolina, USA; Loyola University Chicago, Chicago, Illinois, USA

**Keywords:** recurrent urinary tract infections, urinary microbiome, urobiome, bladder commensals

## Abstract

Here, we present the draft genome sequences of *Lactobacillus delbrueckii* and *Klebsiella pneumoniae*, both isolated from the urinary bladder of an asymptomatic post-menopausal female patient with a diagnosis of recurrent urinary tract infections. These genomes will facilitate analyses of interbacterial interactions in the urinary microbiome.

## ANNOUNCEMENT

Urinary bacterial pathogens have been characterized for decades, while the normal urinary microbiome has only been explored for the last decade with culture-independent sequencing methods and advanced culturing techniques (1, 2). With patient consent and approval of the Duke University Medical Center Institutional Review Board (#Pro00083917), we used Enhanced Quantitative Urine Culture technique to isolate commensal bacteria from the urinary bladder of asymptomatic, post-menopausal female patients with or without diagnosis of recurrent urinary tract infections (rUTI) via aseptic urinary catheterization (1, 3–5).

Here, we report analyses of two bacteria isolated from the same asymptomatic patient with rUTI diagnosis. Both bacterial isolates grew on blood tryptic soy plates upon initial isolation from urine followed by liquid growth in Man Rogosa Sharpe broth at 35°C without agitation (4, 5). These isolates were identified as *Klebsiella pneumoniae* S8 5010–1 and *Lactobacillus delbrueckii* S9 5010–2 by MALDI-TOF mass spectrometry (6). Genomic DNA was isolated (Qiagen UltraClean kit), and sequencing libraries were prepared with Illumina DNA Prep kit. Libraries were sequenced on Illumina NextSeq platform in the 150 bp PE mode. Raw paired-end read sequences (Table 1) were trimmed of adapter and low-quality sequences using the TrimGalore Toolkit (v0.4.4) (7) with default settings, which employs Cutadapt (v1.16) (8). Raw read quality was assessed with FastQC (v0.11.7) (9). Three assemblies were run with the trimmed reads: (i) SPAdes (v3.12.0) in default mode with the “careful” option enabled, (ii) SPAdes with the careful option enabled and a range of kmers (35, 47, 59, 71, 83, 95, 107, 119, and 127), and (iii) Unicycler (v0.4.4) in default mode (10). Final Unicycler assemblies were selected based on their quality and completeness assessment by QUAST (v4.5) (11) and BUSCO (v3.0.2) (12) to identify conserved single-copy orthologs. Command line blastn (BLAST+, v2.7.1) was run in megablast mode for each assembly against the nt database to ensure there is no contamination present (13). The draft genomes were annotated using automated Prokaryotic Genome Annotation Pipeline v3.1 (14). *K. pneumoniae* S8 5010-1 appears to carry three plasmids with two identified via PlasmidFinder 2.1 (15, 16). These plasmids were putatively named pS8_1[IncFIA(HI1) and IncFIB(K)], pS8_2, and pS8_3 (IncFII). Only pS8_2 plasmid (53,643 bp) was circularized during assembly. pS8_2 is not classified into an Inc group by PlasmidFinder. This plasmid does not appear to carry resistance markers but codes for several phage-like proteins based on annotation and Phast search (17). Analysis for the presence of virulence [VirulenceFinder 2.0 (18–20)] and resistance genes [ResFinder4.0 (21, 22)] shows few uropathogen virulence factors (*fimH*, *iutA*, and *traT*) and multi-drug resistance toward at least eight classes of antibiotics including tetracyclines, β-lactams, and amphenicols (18, 21, 23). Preliminary analysis of *L. delbrueckii* S9 5010-2 assembly identified one CRISPR/Cas locus by using the CRISPRCasFinder and putative bacteriocin genes (two helveticin homologues and one enterolysin) by Bagel4 search (24, 25).

**TABLE 1:** Genome characteristics for two commensal urinary bacteria

Assembly features	*K. pneumoniae* S8 5010-1	*L. delbrueckii* S9 5010-2
No. of reads	34,301,320	31,061,372
No. of contigs	126	181
Contig N_50_	257.8 kbp	37.9 kbp
Coverage	953×	2,452×
Completeness [BUSCO (12)]	99.9%	98.2%
Genome size	5.4 Mbp	1.9 Mbp
GC content	57.35%	49.73%

## Data Availability

This whole-genome shotgun project was deposited at DDBJ/ENA/GenBank under the accession numbers JARUKQ000000000 and JARUNW000000000 for *K. pneumoniae* S8 5010-1 and *L. delbrueckii* S9 5010-2, respectively. The raw Illumina sequencing data as sequence read archives are available as SRR24076173 and SRR24078318.
